# Bacteremia Associated With Delayed Cerebral Ischemia After Interventional Treatment of Aneurysmal Subarachnoid Hemorrhage

**DOI:** 10.7759/cureus.100861

**Published:** 2026-01-05

**Authors:** Yukino Irie, Yoshinobu Horio, Kazunori Oda, Mitsutoshi Iwaasa, Koichiro Takemoto, Yoshihiko Nakamura, Hiroshi Abe

**Affiliations:** 1 Neurosurgery, Fukuoka University Faculty of Medicine, Fukuoka, JPN; 2 Emergency and Critical Care Medicine, Fukuoka University Hospital, Fukuoka, JPN

**Keywords:** aneurysmal subarachnoid hemorrhage, bacteremia, cerebral vasospasm, delayed cerebral ischemia, thrombosis

## Abstract

Objective

Delayed cerebral ischemia (DCI) after aneurysmal subarachnoid hemorrhage (aSAH) is a major cause of poor neurological outcomes. Although systemic inflammation related to nosocomial infection has been suggested to contribute to DCI, the specific role of bacteremia remains unclear. This study aimed to evaluate the association between nosocomial infection, particularly bacteremia, and the development of DCI in patients with aSAH treated with coil embolization.

Methods

We retrospectively reviewed patients with aSAH treated at our institution between April 2016 and July 2019. After applying predefined exclusion criteria, including early death due to primary brain injury and insufficient postoperative evaluation, 59 patients who underwent coil embolization were included. Patients were divided into two groups according to the occurrence of DCI. Clinical characteristics, infection-related variables, and outcomes were compared. Univariate analyses were performed, followed by multivariate logistic regression analysis to identify factors associated with DCI.

Results

DCI developed in nine of 59 patients (15%). In univariate analysis, angiographic vasospasm and nosocomial infection were significantly associated with DCI. In multivariate logistic regression analysis, bacteremia was independently associated with the development of DCI (odds ratio, 39.22; 95% confidence interval, 2.25-684.45; P = 0.01).

Conclusions

Bacteremia was independently associated with the development of DCI in patients with aSAH treated with coil embolization. Although causality cannot be established due to the retrospective design and limited sample size, these findings suggest that systemic infection characterized by bacteremia may be clinically relevant to the pathophysiology of DCI. Further prospective studies are warranted to clarify this association.

## Introduction

Aneurysmal subarachnoid hemorrhage (aSAH) is a devastating form of hemorrhagic stroke associated with high morbidity and mortality [1,2]. Despite advances in early aneurysm treatment and neurocritical care, delayed cerebral ischemia (DCI) remains a major cause of secondary brain injury and poor neurological outcome, affecting approximately 30% of patients with aSAH [3].

DCI was initially attributed primarily to angiographic vasospasm. However, accumulating evidence indicates that DCI is a multifactorial process that cannot be explained by large-vessel vasospasm alone [4]. Alternative and complementary mechanisms, including microthrombosis, cortical spreading depolarizations, impaired cerebral autoregulation, microcirculatory dysfunction, and inflammatory processes, have been implicated in the development of delayed ischemic injury after aSAH [5-9].

Several clinical and radiological factors, such as advanced age, neurological severity on admission, the amount of subarachnoid blood, and symptomatic vasospasm, have been reported as predictors of poor outcome after aSAH [10]. Importantly, some prognostic factors develop during hospitalization and may be potentially modifiable [11].

Systemic complications are common during the acute phase of aSAH. In particular, nosocomial infections frequently occur and have been associated with prolonged intensive care unit stay and unfavorable functional outcomes [11-13]. However, previous studies have yielded inconsistent results regarding the relationship between infection and DCI, possibly due to heterogeneity in infection definitions, timing, and outcome measures [11-13]. In contrast, Foreman et al. specifically examined the relationship between nosocomial infection and DCI and reported an association between infectious complications and subsequent DCI development [14].

Acute brain injury, including aSAH, has been shown to induce stroke-related immunosuppression characterized by impaired cellular immune responses, predisposing patients to bacterial infections during hospitalization [15,16]. Bacteremia represents a systemic infectious condition accompanied by pronounced inflammatory and coagulation activation. Experimental and clinical studies have demonstrated that systemic infection can trigger immunothrombosis, a coordinated activation of innate immunity and coagulation leading to microvascular thrombus formation and microcirculatory dysfunction [17,18]. Such mechanisms may theoretically interact with the multifactorial pathophysiology of DCI.

Given these considerations, we conducted a retrospective cohort study to evaluate the association between nosocomial infection, with a particular focus on bacteremia, and the development of DCI in patients with aSAH treated with coil embolization.

## Materials and methods

Study design and patient population

This study was a retrospective observational cohort study conducted at Fukuoka University Hospital, Japan. The study protocol was approved by the Fukuoka University Medical Ethics Review Board (approval number: U21-10-014), and the requirement for informed consent was waived because of the retrospective nature of the study.

Between April 2016 and July 2019, 130 consecutive patients with aSAH were treated at our institution. Among these, 104 patients underwent interventional treatment in the acute phase. To minimize treatment-related heterogeneity, only patients treated with coil embolization were included in the present analysis. Patients who underwent surgical clipping were excluded.

**Figure 1:**
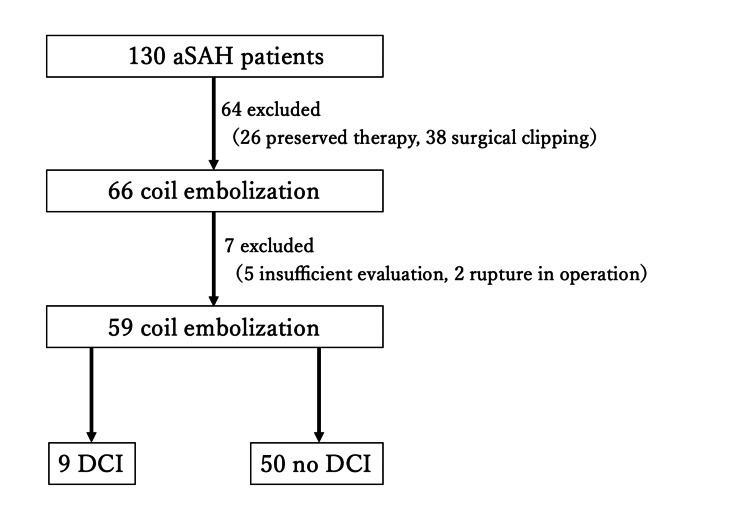
Flowchart of patient selection Between April 2016 and July 2019, 130 consecutive patients with aneurysmal subarachnoid hemorrhage (aSAH) were treated at our institution. Among these, 104 patients underwent interventional treatment in the acute phase. Of these, 66 patients were treated with coil embolization. After excluding patients who died early due to severe primary brain injury and those with insufficient postoperative clinical or imaging evaluation, 59 patients were included in the final analysis. DCI: delayed cerebral ischemia

After applying predefined exclusion criteria, 59 patients were included in the final analysis. Exclusion criteria were as follows: (1) early death due to severe primary brain injury from aSAH, defined as death occurring within a few days of onset before sufficient postoperative or inflammatory evaluation could be performed, and (2) insufficient postoperative clinical or imaging evaluation. The patient selection process is summarized in Figure 1.

Definition of DCI

DCI was defined as new neurological deterioration attributable to cerebral ischemia occurring after aSAH and lasting for at least one hour. Other causes of neurological worsening, including rebleeding, hydrocephalus, electrolyte abnormalities, and central nervous system infection (ventriculitis or meningitis), were excluded by standard imaging, laboratory tests, and physiological assessments.

Neurological deterioration was defined as a decrease of at least 2 points on the Glasgow Coma Scale (GCS). Cerebral ischemia attributable to cerebral vasospasm was included in the definition of DCI. The timing of DCI diagnosis was based on clinical assessment and imaging findings, acknowledging that the exact timing may vary depending on neurological status and imaging availability.

Definition of infection

Infections were defined as bacteremia, pneumonia, or urinary tract infection diagnosed during hospitalization and requiring antibiotic treatment. Central nervous system infections, such as ventriculitis and meningitis, were excluded.

Only infections occurring before the onset of DCI were included in the analysis to minimize the risk of reverse causality. Bacteremia was defined as the presence of pathogenic bacteria in blood cultures. Pneumonia was defined as a new infiltrative shadow on chest radiography or computed tomography, accompanied by clinical signs of infection. Urinary tract infection was defined by positive leukocyte esterase or nitrite on urinalysis and/or bacterial growth on urine culture.

Sepsis was defined as an increase of at least 2 points in the Sequential Organ Failure Assessment (SOFA) score before and after the onset of infection [19].

Evaluation of cerebral vasospasm

Angiographic vasospasm was defined as a reduction of ≥50% in baseline vessel diameter of major cerebral arteries, assessed using digital subtraction angiography, three-dimensional computed tomography angiography, or magnetic resonance angiography. Imaging was performed routinely during days 6-8 and days 14-21 after aSAH, as well as at the onset of neurological symptoms suggestive of vasospasm.

Group classification

Patients were divided into two groups according to the occurrence of DCI: those who developed DCI (DCI group) and those who did not (non-DCI group).

Statistical analysis

Continuous variables are presented as means with standard deviations or medians with interquartile ranges, as appropriate. Categorical variables are presented as counts and percentages. Ordinal clinical scales, including the World Federation of Neurosurgical Societies (WFNS) grade, Hunt and Kosnik grade, and modified Fisher grade, were used descriptively. No predefined cutoff values or thresholds were applied, as these scales do not have established dichotomization criteria.

Comparisons between groups were performed using Student’s t-test or the chi-square test, as appropriate. Univariate analyses were conducted to evaluate associations between clinical variables and the occurrence of DCI. Variables with a P-value < 0.10 in univariate analysis were considered for inclusion in multivariate logistic regression analysis.

To avoid model overfitting due to the limited number of DCI events, multivariate analysis focused on infection-related variables aligned with the primary study objective. Results are presented as odds ratios (ORs) with 95% confidence intervals (CIs). A two-sided P value < 0.05 was considered statistically significant. All statistical analyses were performed using IBM SPSS Statistics for Windows, Version 22 (Released 2013; IBM Corp., Armonk, New York, United States).

## Results

Baseline and clinical characteristics

A total of 59 patients with aSAH treated with coil embolization were included in the analysis. DCI developed in nine patients (15.3%), while 50 patients (84.7%) did not develop DCI.

Baseline and clinical characteristics of patients with and without DCI are summarized in Table 1. Patients who developed DCI were older than those without DCI (mean age, 73.7 ± 6.61 years vs. 60.1 ± 17.97 years; P = 0.03). There was no significant difference in sex distribution between the two groups.

**Table 1:** The baseline characteristics of DCI positive group (Group I) and DCI negative group (Group II) WFNS: World Federation of Neurological Surgeons, aSAH: aneurysmal subarachnoid hemorrhage, SD: standard deviation, mRS: modified Rankin Scale, DCI: delayed cerebral ischemia

Variate	Group I (DCI +)	Group II (DCI -)	Total	P-value
General				
Total number (%)	9 (15.3)	50 (84.7)	59 (100)	-
Sex, male, no (%)	4 (84.1)	18 (36.0)	22 (37.3)	0.637
Age in years, mean±SD	73.7±6.61	60.1±17.97	62.2±17.4	0.03
aSAH grade and outcome				
WFNS grade, mean±SD	4.00±1.32	2.64±1.61	2.85±1.64	0.02
Hunt and Kosnik grade, mean±SD	3.89±1.17	2.74±1.50	2.92±1.50	0.03
Modified Fisher grade, mean±SD	3.89±0.33	2.52±1.26	2.75±1.27	0.002
Discharge mRS, mean±SD	4.22±1.72	2.02±1.81	2.36±1.95	0.001
Major complication				
Angiographic spasm, no (%)	7 (77.8)	3 (6.0)	10 (16.9)	<0.001
Infective complication				
Nosocomial infection, no (%)	5 (55.6)	18 (36.0)	23 (39.0)	0.09
Pneumonia, no (%)	2 (22.2)	10 (20)	12 (20.3)	0.88
Urinary tract infection, no (%)	3 (33.3)	9 (18.0)	12 (20.3)	0.3
Bacteremia, no (%)	2 (22.2)	1 (2.0)	3 (5.1)	0.01
Other complication				
Hydrocephalus, no (%)	0 (0)	4 (8.0)	4 (6.8)	0.39
Takotsubo carditis, no (%)	4 (84.1)	3 (6.0)	7 (11.9)	0.01
Blood analysis				
Max. procalcitonin, mean±SD	17.8±33.2	0.36±0.59	3.02±13.9	<0.001
Max. C-reactive protein, mean±SD	18.0±6.66	8.16±5.08	9.65±6.36	<0.001
Max. white blood cells (10^3^/μL), mean±SD	25.7±24.1	15.0±4.54	16.6±10.6	0.04

Regarding neurological severity and radiological grade on admission, patients in the DCI group tended to present with more severe findings. WFNS grade, Hunt and Kosnik grade, and modified Fisher grade were all higher in patients who developed DCI compared with those who did not. Because these grading scales are ordinal variables, these differences were interpreted descriptively rather than as continuous measures.

Angiographic vasospasm occurred significantly more frequently in the DCI group than in the non-DCI group (77.8% vs. 6.0%; P < 0.001). Functional outcome at discharge, assessed by the modified Rankin Scale, was significantly worse in patients with DCI (mean score, 4.22 ± 1.72 vs. 2.02 ± 1.81; P = 0.001).

With respect to infectious complications, nosocomial infection occurred in 23 patients (39.0%). Although the overall incidence of nosocomial infection did not differ significantly between groups, bacteremia was more frequently observed in the DCI group than in the non-DCI group (22.2% vs. 2.0%; P = 0.01). In contrast, pneumonia and urinary tract infection were not significantly different between the two groups.

Among other complications, takotsubo cardiomyopathy was significantly more common in the DCI group (84.1% vs. 6.0%; P = 0.01), whereas the incidence of hydrocephalus did not differ significantly between groups. Laboratory findings reflecting systemic inflammation were more pronounced in patients with DCI. Peak serum procalcitonin levels, C-reactive protein levels, and white blood cell counts were all significantly higher in the DCI group compared with the non-DCI group.

Univariate analysis

Univariate analyses were performed to evaluate the association between clinical variables and the occurrence of DCI (Table 2). Angiographic vasospasm was significantly associated with DCI (OR, 23.44; 95% CI, 1.75-313.45; P = 0.02). Nosocomial infection was also significantly associated with DCI (OR, 2.24; 95% CI, 1.22-9.19; P = 0.04).

**Table 2:** Univariate and multivariate analysis of factors associated with DCI DCI: delayed cerebral ischemia, OR: odds ratio, CI: confidence interval, WFNS: World Federation of Neurological Surgeons, mRS: modified Rankin Scale, n/a: not applicable

Factor	Univariate analysis			Multivariate analysis		
	OR	95% CI	P-value	OR	95% CI	P-value
WFNS	0.99	0.35-2.76	0.98	n/a	n/a	n/a
Modified Fisher grade	4.04	0.52-31.47	0.18	n/a	n/a	n/a
Discharge mRS	1.12	0.44-2.84	0.81	n/a	n/a	n/a
Angiographic spasm	23.44	1.75-313.45	0.02*	n/a	n/a	n/a
Nosocomial infection	2.24	1.22-9.19	0.04*	0.45	0.05-4.12	0.48
Pneumonia	1.06	0.16-7.11	0.96	n/a	n/a	n/a
Urinary tract infection	1.47	0.23-9.21	0.68	n/a	n/a	n/a
Bacteremia	11.67	0.79-171.71	0.07	39.22	2.25-684.45	0.01*
Max. procalcitonin	2.7	0.87-8.43	0.09	n/a	n/a	n/a
Max. C-reactive protein	1.19	1.00-1.42	0.05	n/a	n/a	n/a
Max. white blood cells	1.00	1.00-1.00	0.99	n/a	n/a	n/a

Among infection subtypes, bacteremia showed a trend toward association with DCI in univariate analysis (OR, 11.67; 95% CI, 0.79-171.71; P = 0.07), whereas pneumonia and urinary tract infection were not significantly associated with DCI.

Multivariate analysis

Variables with a P-value < 0.10 in univariate analysis were considered for inclusion in multivariate logistic regression analysis. Given the limited number of DCI events and to avoid model overfitting, multivariate analysis focused on infection-related variables aligned with the primary study objective.

In multivariate logistic regression analysis, bacteremia remained independently associated with the development of DCI (OR, 39.22; 95% CI, 2.25-684.45; P = 0.01). Other types of nosocomial infection did not show a significant independent association with DCI.

Representative case

A representative case is presented to illustrate the clinical course of bacteremia preceding the onset of DCI. This case is provided for clinical context and does not imply causality.

**Figure 2:**
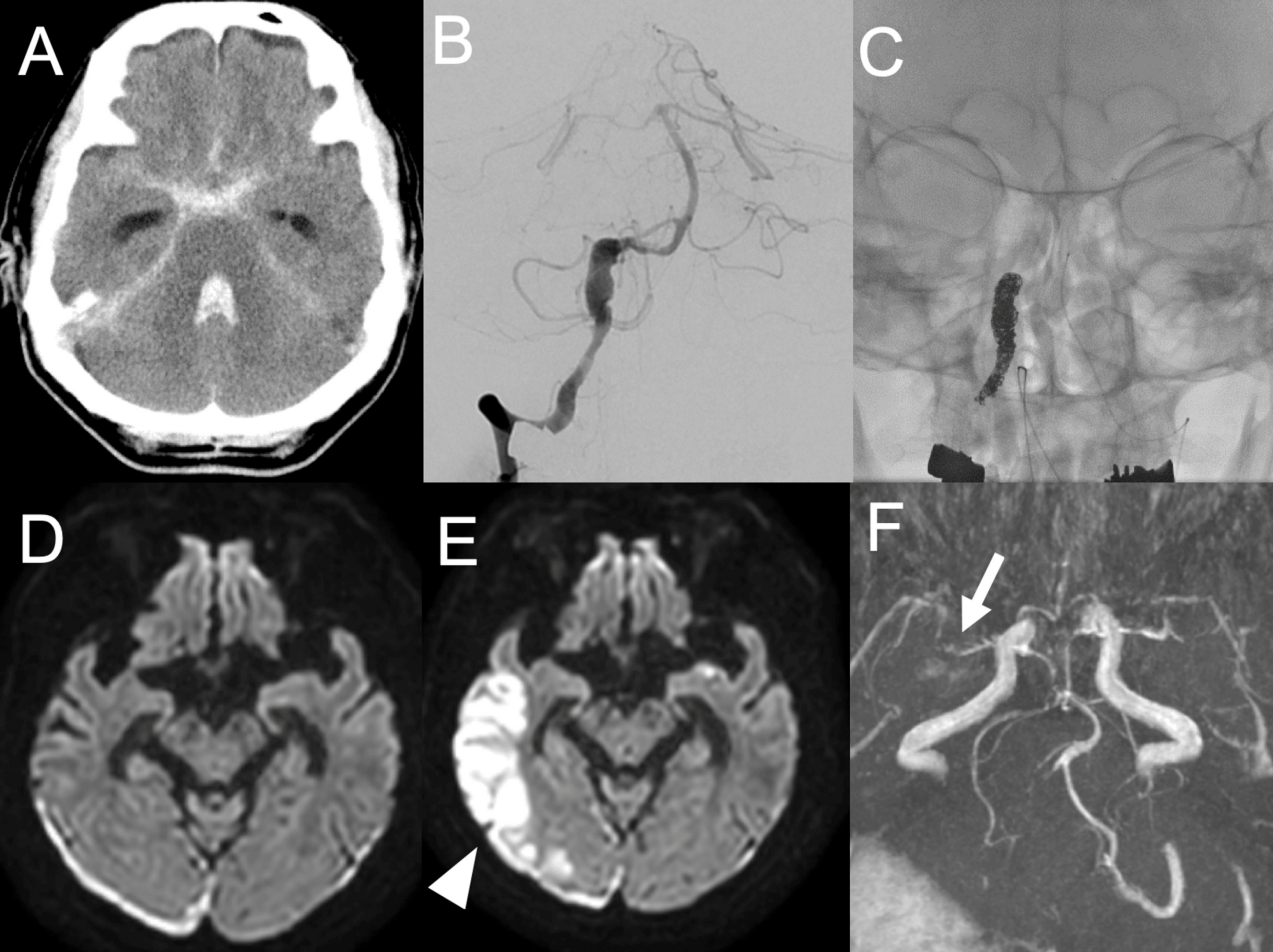
Neuroimaging findings (A) Head computed tomography at onset showing subarachnoid hemorrhage centered in the posterior cranial fossa with intraventricular hemorrhage. (B, C) Cerebral angiography of the right vertebral artery demonstrating irregular spindle-shaped dilatation, consistent with a ruptured dissecting vertebral artery aneurysm; parent artery occlusion was performed. (D) Diffusion-weighted head magnetic resonance imaging on postoperative day 14 showing no fresh infarction, with a high-signal area in the right temporo-occipital subdural region, consistent with subdural hematoma. (E) Diffusion-weighted magnetic resonance angiography of the head on postoperative day 16, after sepsis, demonstrating a fresh infarction in the right temporal lobe (white arrowhead). (F) Head magnetic resonance angiography on postoperative day 14 showing vasospasm in the right middle cerebral artery (white arrow).

A 67-year-old man with aSAH due to a ruptured right vertebral artery dissecting aneurysm underwent endovascular parent artery occlusion on the day of admission (WFNS grade V and modified Fisher grade IV). His neurological status initially improved; however, on day 16 after hemorrhage onset, he developed high-grade fever and subsequent deterioration of consciousness. Blood cultures revealed* Escherichia coli*, and septic shock secondary to urinary tract infection was diagnosed. Diffusion-weighted magnetic resonance imaging demonstrated a new high-signal lesion in the right temporal lobe, and magnetic resonance angiography showed reduced peripheral visualization of the right middle cerebral artery. Despite improvement of systemic inflammation, neurological impairment persisted, and the patient was transferred to a rehabilitation facility with a modified Rankin Scale score of 4 (Figure 2).

## Discussion

In this retrospective cohort study, bacteremia was independently associated with the development of DCI in patients with aSAH treated with coil embolization. While nosocomial infection as a broad category did not retain significance in multivariate analysis, bacteremia emerged as a specific systemic infectious condition associated with DCI.

The pathophysiology of DCI is increasingly recognized as multifactorial. Although angiographic vasospasm has historically been emphasized, multiple studies have demonstrated that vasospasm alone cannot fully account for the occurrence, timing, or distribution of delayed ischemic injury after aSAH [5-9]. Mechanisms such as microthrombosis, cortical spreading depolarizations, impaired autoregulation, and inflammatory activation have been proposed as contributors to DCI, often interacting in a complex manner.

Microthrombosis represents one potential link between inflammation and cerebral ischemia following subarachnoid hemorrhage [5,6]. Pathological and experimental evidence suggests that microvascular thrombosis may impair regional cerebral perfusion independently of large-vessel narrowing. Cortical spreading depolarizations have also been shown to exacerbate ischemic injury through metabolic stress and microvascular constriction [7-9]. Importantly, these mechanisms are not mutually exclusive and may coexist in individual patients.

Systemic infection is known to induce robust inflammatory and coagulation responses. Sepsis and bacteremia trigger activation of innate immunity, endothelial dysfunction, and coagulation cascades, resulting in immunothrombosis and microvascular disturbances [17,18,20]. From a biological perspective, bacteremia may therefore be associated with processes leading to cerebral microcirculatory dysfunction in the vulnerable post-aSAH brain. Elevated inflammatory cytokines after subarachnoid hemorrhage have been reported to correlate with secondary neurological deterioration, supporting a potential role of systemic inflammation in post-aSAH brain injury [21]. However, the present study did not directly measure inflammatory or coagulation biomarkers, and these mechanisms remain speculative.

Acute brain injury has also been shown to induce stroke-related immunosuppression, increasing susceptibility to infection [15,16]. Subarachnoid hemorrhage appears to be associated with particularly pronounced immune dysregulation, which may explain the high incidence of infectious complications observed during the acute phase. In this context, bacteremia may reflect an interaction between brain injury-induced vulnerability and systemic infectious stress rather than an isolated event.

Previous clinical studies evaluating infection after aSAH have primarily focused on functional outcome rather than DCI itself and have reported heterogeneous results [11-13]. Differences in infection definitions, timing relative to neurological deterioration, and outcome measures likely contribute to these inconsistencies. By restricting the analysis to infections occurring before DCI onset and focusing on bacteremia, the present study aimed to reduce temporal ambiguity and isolate a clinically relevant infectious condition.

Several limitations must be acknowledged. First, this was a retrospective, single-center study with a modest sample size and a limited number of DCI events, resulting in wide CIs and limited statistical power. Second, baseline neurological severity differed between groups, and residual confounding cannot be excluded despite multivariate adjustment. Third, inflammatory and coagulation markers were not systematically collected, precluding mechanistic insight. Fourth, the diagnosis of DCI depended on clinical and imaging availability, introducing potential misclassification. Finally, shared vulnerability to both infection and DCI cannot be completely ruled out.

Despite these limitations, the present findings contribute to the evolving concept of DCI as a systemic and multifactorial phenomenon rather than a purely cerebrovascular complication.

## Conclusions

In this retrospective cohort study of patients with aSAH treated with coil embolization, bacteremia was independently associated with the development of DCI. In contrast, localized infections such as pneumonia and urinary tract infection were not significantly associated with DCI.

Although causality cannot be inferred from the present study, these findings suggest that systemic infection characterized by bacteremia may be clinically relevant to the pathophysiology of DCI. Given the exploratory nature of this single-center study and the limited sample size, the results should be interpreted with caution. Further prospective studies with larger cohorts and detailed assessment of inflammatory, coagulation, and hemodynamic factors are warranted to clarify this association and its potential clinical implications.
